# Transition von der Intensivmedizin zur Palliativmedizin: Eine retrospektive Analyse von 102 Konsilanforderungen

**DOI:** 10.1007/s00101-023-01306-z

**Published:** 2023-08-07

**Authors:** Kathrin Lustig, Frank Elsner, Norbert Krumm, Martin Klasen, Roman Rolke, Vera Peuckmann-Post

**Affiliations:** 1 Klinik für Palliativmedizin, Medizinische Fakultät, RWTH Aachen University, Aachen, Deutschland; 2 AIXTRA, Kompetenzzentrum für Training und Patientensicherheit, Medizinische Fakultät, RWTH Aachen University, Aachen, Deutschland; 3 Klinik für Anästhesiologie, Medizinische Fakultät, RWTH Aachen University, Aachen, Deutschland; 4 Institut für Arbeits‑, Sozial- und Umweltmedizin, Medizinische Fakultät, RWTH Aachen University, Aachen, Deutschland

**Keywords:** Intensivmedizin, Symptomkontrolle, Palliativmedizin, Konsildienst, Transition, Intensive care, Symptom management, Palliative care, Consultation team, Transition

## Abstract

**Hintergrund:**

Die Verlegung von Patient:innen der Intensivstation (ITS) auf die Palliativstation (PTS) geht meist mit der Festlegung eines palliativen Konzeptes einher. Die Anpassung der Therapieziele mit primärem Fokus auf Lebensqualität und Symptomkontrolle kann für medizinisches Personal, Patient:innen und Angehörige herausfordernd sein. Beschreibungen dieser Transition sind jedoch rar.

**Fragestellung:**

Ziel der Studie war die Charakterisierung der von den intensivmedizinischen Stationen gestellten Konsile an die Palliativmedizin sowie der Patient:innen durch Beschreibung der Versorgungspfade und Interventionen.

**Methode:**

Retrospektive, quantitative Analyse aller im Jahr 2019 intensivmedizinisch betreuten Patient:innen der Uniklinik RWTH Aachen, für die ein palliativmedizinisches Konsil angefordert worden war.

**Ergebnisse:**

Es wurden *n* = 102 Konsilanfragen der ITS an die Palliativmedizin ausgewertet. Fünf von 32 (16 %) auf die PTS übernommenen Konsilpatient:innen bzw. 35 (34 %) aller 102 Patient:innen konnten in das häusliche Umfeld oder eine stationäre Einrichtung (Pflegeheim, Hospiz, andere) entlassen werden. Die häufigsten Gründe einer Nichtübernahme waren: fehlende Kapazität der PTS (33 %), Versterben auf der Warteliste (20 %) und Ablehnung durch die Patient:innen (20 %). Die durch die Intensivmediziner:innen angefragten konsiliarischen Leistungen „Symptomkontrolle“ (χ^2^ = 10,17; *p* < 0,05) und „Beratung“ (χ^2^ = 12,82; *p* < 0,001) zeigten einen signifikanten Zusammenhang mit der jeweils durch die Palliativmediziner:innen durchgeführten Intervention.

**Schlussfolgerung:**

Der Bedarf palliativmedizinischer Unterstützung der ITS überstieg die Aufnahmekapazität der PTS. Zukünftige Studien sollten Versorgungsmodelle palliativmedizinischer Unterstützung in der Intensivmedizin näher untersuchen.

## Abstract (en)

**Background:**

The transition of patients from the intensive care unit (ICU) to the palliative care (PC) ward often implies changes including establishing a palliative concept. Adaptation of therapeutic goals can be challenging for medical staff, patients and relatives; however, descriptions of these transition trajectories are rare.

**Objective:**

The aim of this retrospective study was to characterize the consultation requests of the ICU to the PC consultation team as well as the patients by a description of trajectories and interventions.

**Methods:**

Retrospective analysis of all patients receiving intensive care at RWTH Aachen University Hospital in 2019 for whom a PC consultation was requested. The patient population transferred from the ICU to the PC ward was compared with the non-transferred population. In each case, the primary consultation was evaluated regarding the following factors: question, vigilance, length of time from consultation request to its performance, and primary focus of the question. The question focus was categorized into “symptom control”, “counselling” and “transfer” (tick options). In addition, a free text field was available for further notes. Exploration of diagnoses was complemented by accessing the electronic health records.

**Results:**

A total of 102 consultation requests from the ICU to the PC ward were evaluated. The morbidity of patients was high, and most patients had at least one of the following diagnoses: pulmonary (62%), cardiovascular (61%), and/or neurological disease (55%). Of the patients 32 (31%) were transferred to the PC ward, among whom weakness (94%), fatigue (77%), anxiety (55%), pain (53%), and dyspnea (48%) were the most frequently noted symptoms. Of the transferred patients 5 (16%) could be discharged to home, nursing home, hospice or other. In total, 35 (34%) of all patients who were seen by palliative care specialists on ICUs in 2019 could be discharged alive. The most frequent reasons for nonadmission were lack of capacity of the PC ward (33%), dying while being on the waiting list (20%), and refusal by the patient (20%). Of the patients, 7 (26%) died within 48 h after they had been transferred to the PC ward. Performed consultation services “symptom control” (χ^2^ = 10.17; *p* < 0.05) and “counselling” (χ^2^ = 12.82; *p* < 0.001), which were requested by the intensive care physicians, showed a significant linkage with the respective intervention performed by the palliative care team. On the other hand, no statistically significant difference was found for requested and performed “transfer” of patients from ICUs to PC ward. Comparing the transferred versus non-transferred patient population, a significantly more frequent transfer of patients with malignant tumors (*p* = 0.00) was observed.

**Conclusion:**

The need for palliative care support in the ICUs exceeded the admission capacity of the PC ward. Future studies should further examine palliative care models in intensive care medicine.

## Hinführung zum Thema

In den letzten Jahren widmeten sich einige Publikationen der Überversorgung und Therapiebegrenzung in der Intensivmedizin [18, 22] und erhöhten hier womöglich das Bewusstsein für die Sinnhaftigkeit der Integration palliativmedizinischer Aspekte in die Intensivmedizin [3, 5, 8, 18, 19, 21–23]. Der Wechsel von einem kurativen zu einem palliativen Konzept bzw. die Integration der Palliativmedizin in die Intensivmedizin stellen dennoch vielschichtige, praktische Herausforderungen für Personal, Patient:innen und Angehörige dar. Konkrete praktische Informationen könnten hier für Transition und Entscheidungsfindungsprozesse in der Intensivmedizin wertvoll sein.

## Ziel der Arbeit

Das Ziel dieser Studie war die Charakterisierung von Patient:innen, Versorgungswegen und Interventionen im Rahmen des Transitionsprozesses von der Intensiv- in die Palliativmedizin. Hierfür wurden die Konsilanforderungen des Jahres 2019 ausgewertet, die von allen intensivmedizinischen Stationen des Uniklinikums an die Klinik für Palliativmedizin der Uniklinik RWTH Aachen gestellt worden waren.

## Methoden

Der palliativmedizinische Konsiliardienst der Klinik für Palliativmedizin führt palliativmedizinische Konsile am Patient:innenbett durch. Das Konsilteam besteht jeweils aus einem Facharzt/einer Fachärztin verschiedener Fachrichtungen sowie einer Pflegekraft. Diese Teammitglieder haben die Zusatzbezeichnung Palliativmedizin (Ärzt:innen) bzw. die Palliative-Care-Ausbildung (Pflegefachpersonen). Bedarfsorientiert werden die Patient:innen auch gemeinsam mit Physiotherapeut:innen, Psycholog:innen und Sozialdienstmitarbeiter:innen visitiert. Gemeinsame multidisziplinäre Teamsitzungen werden zweimal wöchentlich abgehalten, sofern der Behandlungsauftrag über ein einmaliges Konsil (Erstkonsil) hinaus geht. Dabei hat das Konsilteam stets beratende Funktion, sowohl für Mitarbeitende anderer Fachkliniken wie auch gegenüber Patient:innen und Angehörigen. Änderungen der Medikation werden durch die behandelnden Ärzt:innen der jeweils anderen Fachklinik umgesetzt. Bei Aufnahme auf die PTS werden die Symptome der aufzunehmenden Patient:innen entsprechend ihrer Intensität mit einer Skala von 0 (kein) bis 3 (stark) nach Angaben der Patient:innen registriert. Sofern die Patient:innen nicht zu einer Selbsteinschätzung in der Lage sind, wird eine Fremdeinschätzung durch Angehörige oder Pflegende vorgenommen. Diese Skala basiert auf dem für die Palliativmedizin validierten „minimalen Dokumentationssystem für die Palliativmedizin“, „MIDOS“ [27].

**Figure 1:**
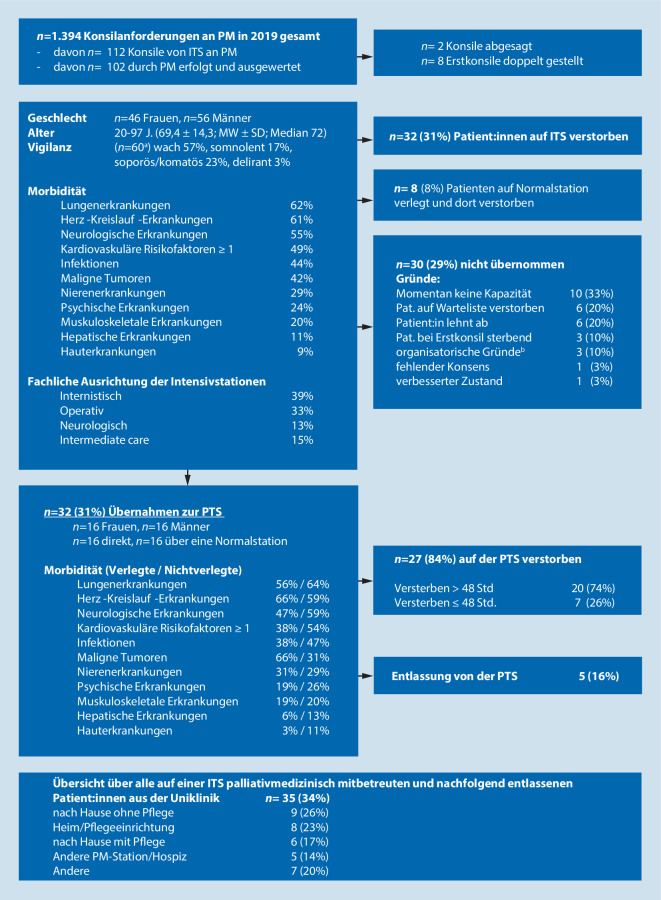

Sämtliche im Jahr 2019 gestellten Konsilanforderungen der Intensivstationen (ITS) an die Klinik für Palliativmedizin der Uniklinik Aachen (UKA) wurden anonymisiert retrospektiv analysiert (Abb. 1). Die Auswertung erfolgte mittels verschiedener statistischer Tests: Der Vergleich der auf die Palliativstation (PTS) verlegten und der nichtverlegten Patient:innen erfolgte mittels *t*-Test für unabhängige Stichproben, der Konsilanforderungen und -durchführung sowie Vigilanz der Patient:innen mittels χ^2^-Test und der Symptomlast nach Übernahme auf die PTS mittels Wilcoxon-Test. Jeweils das erste Konsil wurde hinsichtlich folgender Faktoren ausgewertet: Vigilanz/Allgemeinzustand, Dauer von Konsilstellung bis -durchführung und Fokus der primären Fragestellung. Der Fokus der Konsile wurde kategorisiert in: Symptomkontrolle, Beratung und Übernahme (Ankreuzoptionen). Zudem war in den Konsilanforderungen ein Freitextfeld für Kommentare vorhanden; Mehrfachnennungen waren möglich.

Der Allgemeinzustand wurde anhand der beschriebenen Vigilanz (z. B. „wach“ oder „somnolent“) und des Barthel-Indexes zum Zeitpunkt des ersten Konsils (± 1 Tag) registriert, entsprechend der Dokumentation der Palliativmediziner:innen. Die Erhebung der Diagnosen erfolgte aus den Verlege- oder Entlassungsbriefen.

Die Symptome der auf die PTS übernommenen Patient:innen wurden erfasst (Intensität-Score 0–3). Die von der ITS auf die PTS verlegten Patient:innen wurden mit den nichtverlegten Patient:innen verglichen.

Die Erhebung der Daten erfolgte mithilfe der Dokumentation in der Krankenhaussoftware „Medico“. Dort hinterlegte Arztbriefe ermöglichten eine ergänzende Dokumentation vorbekannter Diagnosen. Die Daten wurden in Excel (Microsoft, USA, Microsoft Office Professional Plus 2016) erfasst und anschließend mit der Statistiksoftware IBM SPSS Statistics 25 (IBM Corp., Armonk, NY, USA) ausgewertet. Die Ethikkommission der Medizinischen Fakultät der RWTH Uniklinik Aachen sah bei einer „Bitte um Stellungnahme“ „keine ethischen oder berufsrechtlichen Bedenken“ (EK 382/19).

## Ergebnisse

Abb. 1 zeigt die Versorgungspfade und Charakteristika der gesamten Konsilpopulation. Im Jahr 2019 wurden innerhalb des UKA insgesamt 1394 Konsile an die Palliativmedizin gestellt, davon gab es 112 Anfragen von ITS, von denen 102 als stattgefundene Erstkonsile analysiert werden konnten. Die meisten Konsilanforderungen (39 %) wurden von internistisch geführten Intensivstationen gestellt.

Die Dauer des stationären Aufenthaltes der Patient:innen auf der ITS bis zur ersten Konsilanfrage betrug im Mittel 13 Tage (Median = 7; Interquartilsabstand = 12; *n* = 92). Die Dauer zwischen der Konsilstellung und der Durchführung des Konsils betrug 0,4 ± 1,2 Tage (*n* = 91). Insgesamt verstarben 67 Patient:innen (66 %) der 102 Patient:innen während ihres Aufenthalts im UKA. Die im Krankenhaus verstorbenen Patient:innen lebten nach dem Primärkonsil noch 8,1 Tage (Median: 4; Interquartilsabstand = 9; *n* = 62). In 6 Fällen wurde angegeben, dass Patient:innen nicht auf die PTS verlegt werden konnten, da sie zuvor verstarben.

Von der gesamten Konsilpopulation wurden 35 Patient:innen (34 %) aus dem UKA entlassen, die meisten davon in das häusliche Umfeld. Die Dauer zwischen Primärkonsil und Entlassung betrug 18,8 Tage (Median = 11,5; Interquartilsabstand = 18; *n* = 32).

### Konsile

Die Morbidität der Konsilpopulation war hoch (Abb. 1). Intensivmediziner:innen baten konsiliarisch überwiegend um Beratung (62 %), gefolgt von Übernahme (54 %) und Symptomkontrolle (30 %). Hingegen dokumentierten die Palliativmediziner:innen zwar ähnlich häufig Beratung (64 %), jedoch häufiger Symptomkontrolle (55 %) und empfahlen seltener als angefragt eine Übernahme (25 %) als konsiliarische Leistung.

Die durch die Intensivmediziner:innen angefragten konsiliarischen Leistungen „Symptomkontrolle“ (χ^2^ = 10,17; *p* < 0,05) und „Beratung“ (χ^2^ = 12,82; *p* < 0,001) zeigten einen signifikanten Zusammenhang mit der jeweils durch die Palliativmediziner:innen durchgeführten Intervention. Bei der Intervention „Übernahme“ hingegen zeigte sich kein Zusammenhang zwischen konsiliarischen Anforderungen und Durchführungen (χ^2^ = 1,85; *p* = 0,17).

Bei 52 von 102 (51 %) der Patient:innen der Gesamtpopulation stellten die Palliativmediziner:innen die Notwendigkeit einer Symptomkontrolle fest. Zu den häufigsten Symptomen zählten bei den auf die PTS übernommenen Patient:innen Schwäche 94 % (bei *n* = 29 von 31 dokumentierten Patient:innen), Müdigkeit 77 % (24/31), Angst 55 % (11/20), Schmerz 53 % (16/30) und Luftnot 48 % (15/31).

### Vergleich verlegter und nichtverlegter Patient:innen

Von den 32 auf die PTS übernommenen Patient:innen wurden 16 direkt und 16 erst nach einer Zwischenverlegung auf eine andere Station zur PTS übernommen. Bei 83 Patient:innen wurde die Vigilanz in der Konsildokumentation beschrieben, war jedoch nur bei 60 Patient:innen eindeutig folgenden Kriterien zuzuordnen: 34 (57 %) wach, 10 (17 %) somnolent, 14 (23 %) nicht ansprechbar (soporös/komatös) und 2 (3 %) delirant. Der Barthel-Index wurde dokumentiert für 44 Patient:innen und zeigte ein sehr niedriges Maß für alltägliche Fähigkeiten an (Range 0–50 von maximal möglichem Wert 100 (100: komplette Selbstständigkeit); MW 11,82 ± SD 13,7).

**Figure 2:**
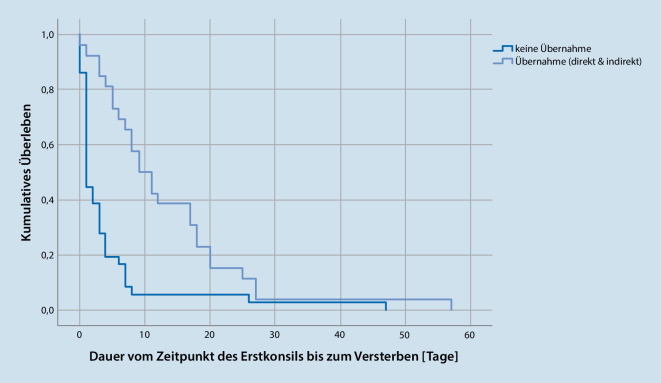

Die übernommenen Patient:innen unterschieden sich von den nichtübernommenen Patient:innen nicht bezüglich ihres Alters (mittleres Alter Übernahme: 71 Jahre; Nichtübernahme: 69 Jahre; t(100) = 0,62, *p* = 0,27) jedoch fanden sich folgende signifikante Unterschiede: Übernommene Patient:innen waren häufiger wach (*p* < 0,05), wiesen häufiger ein Tumorleiden auf (*p* < 0,001) und lebten länger als die nichtübernommenen Patient:innen (*p* < 0,001; Abb. 2). Alle weiteren Diagnosen wiesen keine signifikanten Unterschiede zwischen übernommenen und nichtübernommenen Patient:innen auf.

Bei den auf die PTS aufgenommenen Patient:innen konnte innerhalb der ersten 24 h nach der Übernahme keine Verbesserung der Symptomkontrolle festgestellt werden. Die empfundene Schwäche der Patient:innen nahm signifikant zu (Wilcoxon-Test bei verbundenen Stichproben; W(standardisiert) = 2,35; *p* < 0,05).

Die Gründe der Nichtübernahme gingen in 30 Fällen aus der schriftlichen Beantwortung der Konsilanfrage durch die Palliativmediziner:innen hervor (Abb. 1). Zehn Patient:innen (33 %) konnten aufgrund unzureichender Kapazitäten nicht auf die PTS verlegt werden.

## Diskussion

Die vorliegende Studie hatte zum Ziel, Versorgungspfade intensivmedizinscher Patient:innen sowie Interventionen im Übergang zur Palliativmedizin mithilfe retrospektiver Analyse der erfolgten Konsile zu beschreiben. Die Konsilpatient:innen wiesen eine hohe Morbidität auf. Es konnte gezeigt werden, dass ein Drittel (34 %) der palliativmedizinisch mitbetreuten ITS-Patient:innen im Verlauf aus der Uniklinik entlassen werden konnte. Konsilanforderungen und erbrachte palliativmedizinische Leistungen bezüglich Symptomkontrolle und Beratung wiesen einen signifikanten Zusammenhang auf. Dieser Befund bestätigt eine angemessene interdisziplinäre Zusammenarbeit.

Wenn bei Patient:innen auf der ITS ein Therapiewechsel von kurativen Zielen zu primär symptomlindernden, palliativen Maßnahmen erfolgt, hat sich die Unterstützung durch palliativmedizinische Teams etabliert [8, 9]. Die Einbindung von Palliativmedizin kann zu weniger intensivmedizinischen Aufnahmen und kürzerer Liegedauer sowie verbesserter Lebensqualität, Behandlungsqualität und Versorgung bei reduzierten Kosten führen [2, 7, 8, 20]. Darüber hinaus können palliativmedizinische Teams gezielt bei herausfordernden und zeitintensiven Gesprächen unterstützen, z. B. im Rahmen von Familienkonferenzen oder im Umgang mit kognitiv stark beeinträchtigten Patient:innen.

### Integration der Palliativmedizin: früh- versus rechtzeitig

Temel et al. statuierten ein Exempel für die frühzeitige Integration von Palliativmedizin, die in ihrer Studie zu Patient:innen mit nichtkleinzelligem Lungenkarzinom (NSCLC) im primären Outcome eine Verbesserung der Lebensqualität sowie Reduktion von Angst und Depression in der Interventionsgruppe zeigen konnte. In der Sekundäranalyse zeigte sich sogar ein erhöhtes Überleben [29]. Während Studien zum „Early-integration“-Konzept die Palliativmedizin regelhaft „frühzeitig“ ab der Diagnosestellung hinzuzogen, empfahlen Hui und Bruera einen personalisierten Ansatz eines „rechtzeitigen“ („timely“) Hinzuziehens der Palliativmedizin [15]: Dieser beinhaltet eine Integration der Palliativmedizin erst bei zeitlichem, individuellen Bedarf der Patient:innen, sodass wenig vorhandene palliativmedizinische Ressourcen gezielt genutzt werden können. Als Voraussetzungen identifizieren die Autoren 4 Schlüsselkomponenten: 1) systematisches Routinescreening, 2) definierte Überweisungskriterien, 3) Mechanismen zur Überweisungstriggerung geeigneter Patient:innen und 4) eine palliativmedizinische Ambulanz mit angemessener personeller Ausstattung.

Im Hinblick auf unsere Studie erfolgte die Stellung des Konsils seitens der ITS jedoch im Mittel nach 13 Tagen, insgesamt 12 Patient:innen waren beim Erstkonsil sterbend/verstarben auf der Warteliste, und 7 von der ITS auf die PTS übernommene Patient:innen verstarben innerhalb von 48 h. Sowohl das Hinzuziehen der Palliativmediziner:innen zu fortgeschrittenem Zeitpunkt als auch die begrenzte Kapazität der PTS könnten diesen Beobachtungen zugrunde gelegen haben. Nelson et al. erwähnen die Sorge von Ärzt:innen, dass das Hinzuziehen von Palliativmedizin zwangsläufig zu Therapiezieländerungen führen könnte, was eine verzögerte Konsilstellung an die Palliativmedizin ebenfalls erklären könnte [24].

Zukünftig wäre es wünschenswert, wenn die Integration palliativmedizinischer Unterstützung mit einer zeitgerechten Konsilstellung beginnt. Denkbar wäre ein systematisches Routinescreening hinsichtlich eines palliativmedizinischen Unterstützungsbedarfes, wie in der Literatur vorgeschlagen [15]. Screening könnte standardisiert bereits bei Aufnahme in ein Krankenhaus erfolgen sowie bei Aufnahme auf eine ITS. So könnten Patient:innen bereits vor einem (potenziellen) Intensivstationsaufenthalt zeitnah beraten, eine multidisziplinäre Zusammenarbeit koordiniert und medizinisches Personal bei der Symptomkontrolle unterstützt werden.

### Palliativversorgung in der interdisziplinären Intensivversorgung: mehr als nur „Entscheidungsfindung am Lebensende“

In dieser Studie konnte verdeutlicht werden, dass die Konsilanforderungen nicht auf Therapieentscheidungen im Sterbeprozess begrenzt waren. Tatsächlich wurden insgesamt 34 % der palliativmedizinisch mitbetreuten und 16 % der auf die PTS übernommenen Konsilpatient:innen in das häusliche Umfeld, Hospiz, Pflegeheim oder andere PTS entlassen. Teilweise konnten durch konsiliarische Mitbetreuung des palliativmedizinischen Teams spezielle individuelle Lösungen gefunden werden, die eine Entlassung in das häusliche Umfeld ermöglichten bzw. harmonisch gestalteten. Das Finden dieser Lösungen erfordert in der Regel ausführliche Gespräche im Familienkreis, gelegentlich auch mit Moderation im Falle von Konflikten unter Angehörigen. Diese Interventionen können daher zeitaufwendig sein und möglicherweise nur begrenzt auf intensivmedizinischen Stationen durchgeführt werden.

Schuster et al. [28] fassen in ihrem Review zu „palliativen Therapiekonzepten in der Intensivmedizin“ zusammen, dass „Intensivmediziner palliativmedizinische Behandlungsgrundsätze kennen und als Ressource einer multiprofessionellen Versorgung begreifen“ sollten. Sie empfehlen neben der Verbesserung von Symptomkontrolle und Beratung von Patient:innen und Angehörigen die „Etablierung strukturierter interdisziplinärer und interprofessioneller Kommunikationsstrukturen“, auch um einen interdisziplinären Konsens bei der Erreichung individueller Therapieziele erfolgreich zu bahnen.

Adler et al. untersuchten die Akzeptanz von publizierten Triggerfaktoren [1] zur Identifizierung von palliativmedizinischem Bedarf auf der Intensivstation. Die Autoren identifizierten 6 Faktoren: „Anfrage durch Patient“, „Anfrage durch Familie“, „keine kurative Optionen“, „hohe Symptomlast“, „nichtoperable Malignität“ und „abgelehntes kuratives Konzept“. Die Autoren hoben hervor, dass die meisten Trigger-Faktoren [2, 4, 25] von den befragten Intensivmediziner:innen nicht akzeptiert worden waren, obwohl für die Mehrzahl dieser Faktoren laut Literatur Validität für die Intensivmedizin nachgewiesen worden war. Die Autor:innen konstatierten eine Diskrepanz zwischen Evidenz und klinischer Praxis [1]. Eine mögliche Erklärung für die Diskrepanz könnte ein unterschiedliches Verständnis von „Palliativmedizin“ sein. Die Autor:innen einer konsensusbasierten Definition stellten fest, dass die größte Herausforderung darin lag, eine gemeinsame Ebene von denjenigen, die Palliativmedizin als „Lindern allen Leids“ verstehen, mit denjenigen zusammenzubringen, für die Palliativmedizin stets eine stark verkürzte Lebenszeit betrifft [26]. Möglicherweise assoziierten viele Intensivmediziner:innen mit einer Palliativversorgung eine „Betreuung in den letzten Lebenstagen“, während die Palliativmedizin selbst auch zunehmend nach einer „frühen Integration“ strebt und selbstverständlich viele Patient:innen wieder von einer PTS aus beispielsweise nach Hause oder in eine hospizliche Umgebung entlassen werden.

Obwohl Verbesserungen durch Palliativmedizin zahlreich belegt sind, und sogar Behandlungskosten gesenkt werden können [2, 7, 8, 20], ist die Palliativmedizin bis dato immer noch nicht als feste Struktur in die Patient:innenversorgung integriert. Bruera schlägt als zukünftigen Fokus palliativmedizinischer Forschung die Untersuchung der Frage vor, warum Personen in Führungspositionen („clinical and academic leaders“) bislang nicht in eine robuste palliativmedizinische Versorgung investiert haben [15]. Schließlich hebt er hervor, dass wir alle irgendwann von Leid bzw. Sterben betroffen sein werden, auch die Personen in Führungspositionen.

### Lösungswege für integrierende Ansätze zwischen Intensiv- und Palliativmedizin

Ein Lösungsansatz zur Optimierung der interdisziplinären Zusammenarbeit zwischen Intensiv- und Palliativmedizin ist die Einführung einer Kombination eines konsultativen und eines integrativen Modells. Bei einem solchen Mischmodell gibt es sowohl einen festen Zeitraum, in dem Palliativmediziner:innen auf einer ITS anwesend sind, als auch die Möglichkeit, unabhängig von diesen Zeiten Konsilanfragen zu stellen [9]. Individuell an Bedürfnissen und Therapiezielen der Patient:innen orientiert, könnte so das Ausmaß der palliativmedizinischen Unterstützung bestimmt werden: Primär geführt von Palliativmediziner:innen auf einer PTS bzw. palliativmedizinisch begleitend in einem primär intensivmedizinischen Setting. Die begrenzte Aufnahmekapazität einer palliativmedizinischen Station, welche auch in der vorliegenden Studie ein limitierender Faktor war, könnte womöglich durch in der Literatur bereits beschriebene Versorgungsmodelle kompensiert werden [16]. Wenn man bedenkt, dass jeder vierte Patient, der im Krankenhaus verstirbt, zuvor intensivmedizinisch behandelt wird, [10] so erscheint dieser Aspekt sehr relevant.

Die Rolle der Pflege als Teil eines palliativmedizinischen Konsildienstes scheint unterschiedliche Gewichtung zu finden. Eine Unterstützung von Pflegenden für Pflegende wäre auch als anzufordernde Konsilleistung denkbar und könnte eine wichtige Ressource im klinischen Alltag darstellen. Mögliche Anforderungsgründe für pflegerische Unterstützung könnten sein: Anleitung zur Mundpflege bei Durstgefühl, Unterstützung beim Aufziehen von Perfusoren für hochpotente Opioide (die auf einer bestimmten Station nicht üblich sind) oder schlichtweg das Vermitteln pflegerischer „Kniffe“ im palliativmedizinischen Bereich.

### Charakteristika und Verlauf von in die Palliativmedizin übernommenen ITS-Patient:innen

Im Hinblick auf die übernommenen Patient:innen wurden solche mit einer malignen Tumorerkrankung signifikant häufiger als Patient:innen mit anderen Erkrankungen übernommen. Dies kann verschiedene Gründe haben: Es ist möglich, dass von den anfordernden Kolleg:innen bei diesen Patient:innen mehr Unterstützung nötig bzw. möglich war als bei anderen Erkrankungen. Zum anderen ist die Zusammenarbeit zwischen Onkologie und Palliativmedizin traditionell gewachsen und inzwischen mit genügend Evidenz für eine bessere Versorgung der Patienten belegt [16, 19]. Somit existiert in der Palliativversorgung traditionell eine größere Vertrautheit mit den Bedürfnissen und Wünschen onkologischer Patient:innen.

Weiterhin profitierten in einer aktuellen Studie insbesondere Patient:innen mit onkologischen Erkrankungen von leitliniengerechter Palliativmedizin, nicht jedoch Patient:innen mit nichtonkologischen Erkrankungen [13]. Zukünftige Ausbildungskonzepte sollten daher beinhalten, dass auch Patient:innen mit nichtonkologischen Erkrankungen von der Palliativmedizin profitieren können und sie diesen zugänglich sein soll.

Eine Besserung der Symptomatik durch die palliativmedizinischen Interventionen nach Übernahme konnte in dieser Studie, im Gegensatz zu anderen Studien [19], nicht festgestellt werden. Ein Grund ist die teilweise fehlende bzw. nichteinheitliche Dokumentation von Symptomen und Funktionsstatus, was ein international bekanntes Phänomen in der Public-Health-Literatur darstellt [6]. In Zukunft ist eine ausführlichere und konsequentere standardisierte Dokumentation wünschenswert, damit retrospektiv eine fundierte Auswertung des Nutzens der palliativmedizinischen Maßnahmen erfolgen kann [19]. Auch aus wissenschaftlicher Perspektive wäre daher eine gut vernetzte Palliativmedizin, womöglich als übergeordnete Struktur, wertvoll [16].

Abschließend bleibt festzustellen, dass sich ein seit Jahren zunehmender Trend einer intensivmedizinischen Behandlung am Lebensende zeigt [10]. Während es allgemein in der Bevölkerung der häufigste Wunsch ist, am Lebensende zu Hause zu sein, sterben tatsächlich bis zu 50 % der Menschen in einem Krankenhaus [11, 12]. Diese Studie könnte ein weiterer Impuls für die Integration palliativmedizinischer Versorgungskonzepte in der Intensivmedizin sein.

## Limitationen

Der Charakter einer retrospektiven Untersuchung lässt nur eine Beschreibung der Daten zu. Die Einschätzung der zu übernehmenden Patient:innen mit einer größeren Wachheit und Überlebenszeit war assoziiert mit einem längeren Überleben (Abb. 2). Das signifikant längere Überleben der auf die PTS übernommenen Patient:innen darf keinesfalls isoliert betrachtet werden. Eine naheliegende Erklärung ist die bewusste Auswahl der signifikant vigilanteren Patient:innen zur Übernahme durch die Palliativmediziner:innen aufgrund der klinischen Einschätzung. Darüber hinaus wurden sich unmittelbar im Sterbeprozess befindliche Patient:innen nicht übernommen, sondern in der jeweiligen Fachkliniken konsiliarisch mitbetreut. Gründe für eine Entscheidung zur Übernahme der vigilanteren Patient:innen wurden nicht dokumentiert.

Die Symptomlast sowie körperliche Funktion (z. B. Barthel-Index) der konsiliarisch gesehenen Patient:innen wurden nicht standardisiert erhoben, sodass aufgrund fehlender Daten ein Vergleich nicht möglich war. Datenverlust bei der Outcome Erhebung in der Palliativmedizin ist eine bekannte Herausforderung [17]. Ein darüber hinaus nahender Sterbeprozesses des Großteils der auf die PTS übernommenen Patient:innen kann erklären, dass keine Verbesserung der Symptomlast nachweisbar war, denn eine hohe Symptomlast sowie ein sich verschlechternder Allgemeinzustand werden regelhaft im Sterbeprozess beobachtet [14].

Die Art der Dokumentation, bei der zu einem beliebigen Zeitpunkt des Tages die aktuelle Symptomlast auf eine Skala von 0 bis 3 notiert wird, könnte außerdem zu unscharf sein, um kleine Effekte zu identifizieren. Erschwert wird die Erfassung von Symptomen darüber hinaus durch die Notwendigkeit einer Fremdeinschätzung durch das medizinische Personal für Patient:innen, die selbst keine Angaben zu Symptomen machen konnten. Patient:innen, bei denen Angaben für eine Berechnung fehlten, wurden aus der jeweiligen Berechnung ausgeschlossen.

## Fazit für die Praxis

- Ein Drittel der Konsilpopulation konnte in das häusliche Umfeld, Hospiz, Pflegeheim oder andere palliativmedizinisch geführte Station entlassen werden. Intensivstationen können von palliativmedizinischer Expertise bezüglich Symptomkontrolle und Versorgungsplanung profitieren.
- Zukünftige Versorgungskonzepte sollten den Fokus auf eine robuste Zusammenarbeit von Intensivmedizin und Palliativmedizin legen. Dabei sollten konsultative vs. integrative Konzepte sowie ein früh- vs. rechtzeitiges Hinzuziehen der Palliativmedizin beleuchtet werden.
- Systematisches Screening zur Identifizierung von palliativmedizinischem Bedarf sollte etabliert werden, z. B. bereits bei Aufnahme in ein Krankenhaus bzw. auf eine ITS. Sinnvolle Kriterien hierfür sollten in künftigen Studien herausgearbeitet werden (z. B. Alter, Morbidität und häufige stationäre Aufenthalte). Auch Patient:innen mit nichtonkologischen Erkrankungen sollten Zugang zur Palliativmedizin haben.
- Standardisierte Dokumentation im Klinikalltag ist wichtig, um Outcomes vergleichen und die Qualität spezieller Interventionen überprüfen zu können. Hier sind zukünftig Fachgesellschaften und nationale Strategien gefordert, ein einheitliches Vorgehen anzustreben.
